# Molecular probe technology detects bacteria without culture

**DOI:** 10.1186/1471-2180-12-29

**Published:** 2012-03-09

**Authors:** Richard W Hyman, Robert P St Onge, Hyunsung Kim, John S Tamaresis, Molly Miranda, Ana Maria Aparicio, Marilyn Fukushima, Nader Pourmand, Linda C Giudice, Ronald W Davis

**Affiliations:** 1 Departments of Biochemistry, Stanford University, Stanford, CA, USA; 2 Departments of Biochemistry and Genetics, Stanford University, Stanford, CA, USA; 3 Stanford Genome Technology Center, Stanford University, Stanford, CA, USA; 4 Biomolecular Engineering Department, Jack Baskin School of Engineering, University of California, Santa Cruz, Santa Cruz, CA, USA; 5 Laboratory of Endometrial Biology, Functional Genomics, and Stem Cell Research, Department of Obstetrics, Gynecology, and Reproductive Sciences, University of California, San Francisco, San Francisco, CA, USA; 6 Department of Obstetrics, Gynecology, and Reproductive Sciences, University of California, San Francisco, San Francisco, CA, USA; 7 Stanford Genome Technology Center, 855 S. California St., Palo Alto, CA 94304, USA

## Abstract

**Background:**

Our ultimate goal is to detect the entire human microbiome, in health and in disease, in a single reaction tube, and employing only commercially available reagents. To that end, we adapted molecular inversion probes to detect bacteria using solely a massively multiplex molecular technology. This molecular probe technology does not require growth of the bacteria in culture. Rather, the molecular probe technology requires only a sequence of forty sequential bases unique to the genome of the bacterium of interest. In this communication, we report the first results of employing our molecular probes to detect bacteria in clinical samples.

**Results:**

While the assay on Affymetrix GenFlex Tag16K arrays allows the multiplexing of the detection of the bacteria in each clinical sample, one Affymetrix GenFlex Tag16K array must be used for each clinical sample. To multiplex the clinical samples, we introduce a second, independent assay for the molecular probes employing Sequencing by Oligonucleotide Ligation and Detection. By adding one unique oligonucleotide barcode for each clinical sample, we combine the samples after processing, but before sequencing, and sequence them together.

**Conclusions:**

Overall, we have employed 192 molecular probes representing 40 bacteria to detect the bacteria in twenty-one vaginal swabs as assessed by the Affymetrix GenFlex Tag16K assay and fourteen of those by the Sequencing by Oligonucleotide Ligation and Detection assay. The correlations among the assays were excellent.

## Background

The Human Microbiome Project has taken a metagenomic approach to identifying the bacteria in a wide variety of sites on and in the human body because the substantial majority of these bacteria have not been grown in culture [*e.g*.,[1]. Second generation DNA sequencing on this level presents a formidable informatics challenge. It is unlikely that such sequencing will be useful for individual investigators and clinical diagnostics. Therefore, the challenge is to detect each bacterium in a mixture when all that is known about the bacterium is a partial genome sequence.

In a previous publication [2], we presented our adaption of molecular inversion probes [MIP; [3] to detect bacteria using a massively multiplex molecular technology. MIP technology was developed, in large part, to discover and assay single nucleotide polymorphisms in human DNA [4]. The human genome is diploid. Bacterial genomes are haploid, and, therefore, the background for molecular probe technology is significantly lower. Because of this important difference, we simplified the method by dispensing with the "inversion".

Our method requires only a sequence of forty sequential bases unique to the bacterial genome of interest, such as derived from the sequences produced by the Human Microbiome Project. All necessary reagents are commercially available, including an Affymetrix GenFlex Tag16K array v2 (Tag4 array). Whereas we reported previously the results of employing the molecular probes with simulated clinical samples (artificial mixtures of bacterial genomic DNAs made to resemble clinical samples), we report now the results with a set of clinical samples: swabs of the vaginal epithelium.

While the assay on Tag4 arrays allows the multiplexing of the detection of the bacteria in each clinical sample, nevertheless, one Tag4 array must be used for each sample. To multiplex the clinical samples, we introduce a second, independent assay for the molecular probes employing Sequencing by Oligonucleotide Ligation and Detection (SOLiD). All reagents are also commercially available. By adding one unique oligonucleotide barcode for each clinical sample, we combine the molecular probes after processing each sample, but before sequencing, and SOLiD sequence them all together. Overall, we have employed 192 molecular probes representing 40 bacteria to detect the bacteria in twenty-one vaginal swabs as assessed by the Tag4 assay and fourteen of those by the SOLiD assay.

## Results

**Figure 1:**
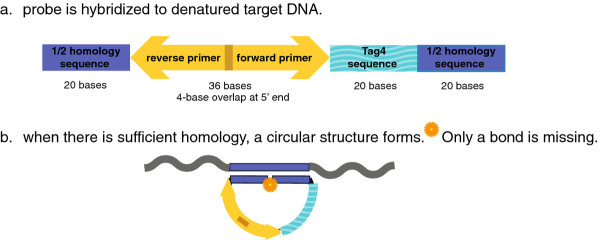
**Molecular probe design**. (**a**) The deep blue color represents the 40-base sequence similarity domain (the Homer), which is divided into two 20-base segments. The aquamarine color represents the 20-base oligonucleotide barcode from the Tag4 array. The yellow color represents the 36-base domain for the two 20 base PCR primers. The two 20 base primers overlap by 4 bases at the 5' ends. The total length is 96 bases. The 5' end is phosphorylated. (**b**) The molecular probe mixture is incubated with the denatured target DNA under annealing conditions. Where sufficient sequence similarity exists between the molecular probe and the target single-stranded DNA (indicated by the deep blue color), 40 bp of duplex DNA are formed. The 5'-phosphorylated end of the molecular probe is adjacent to the 3'-hydroxyl end of the probe with no bases missing.

We have published the design of our molecular probes (Figure 1a) and our assay procedure [2]. These are recapitulated in the Methods section.

### Simulated clinical samples

Our earlier work with simulated clinical samples proved critical for development of the molecular probe technology as assayed on Tag4 arrays [2]. Therefore, we employed the same simulated clinical samples and assayed them by SOLiD sequencing. Table 1 presents the results. When assayed by SOLiD sequencing (Table 1), there were no false negatives and one false positive. Importantly, *Lactobacillus acidophilus *was correctly found in SCA. With further regard to *Lactobacillus *for the five simulated clinical samples, the molecular probes for *L. brevis *were positive for only SCC, the sole sample containing *L. brevis*. The *L. gasseri *probes were positive for the three simulated clinical samples containing *L. gasseri *(SCB, SCC, SCE) and falsely positive for one more (SCA). Although not shown explicitly in Table 1, we counted a large number of bacteria correctly negative for each of the five simulated clinical samples: SCA, 31 correct negatives; SCB, 33; SCC, 33; SCD, 32, SCE, 34.

**Table 1:** The composition of the five simulated clinical samples and the detection of bacteria in each

Genome/ Mixture	A			B			C			D			E		
	
	1	2	3	1	2	3	1	2	3	1	2	3	1	2	3
*A. baumannii*	1.75	**1**	**1**		0	0	3.5	**1**	**1**	3.5	**1**	**1**		0	0
*B. fragilis*		0	0	1.8	**1**	**1**		0	0	1.8	**1**	**1**		0	0
*B. longum*	10.0	**1**	**1**		0	0		0	0		0	0		0	0
*E. coli*	2.25	**1**	**1**		0	0		0	0		0	0	0.45	**1**	**1**
*L. acidophilus*	10.0	0	**1**		0	0		0	0		0	0		0	0
*L. brevis*		0	0		0	0	10.0	**1**	**1**		0	0		0	0
*L. gasseri*		0	**1**	10.0	**1**	**1**	1.6	**1**	**1**		0	0	1.6	**1**	**1**
*S. aureus*		0	0	2.2	**1**	**1**		0	0	10.0	**1**	**1**		0	0
*S. agalactiae*		0	0	2.4	**1**	**1**		0	0	10.0	**1**	**1**		0	0
*T. pallidum*	0.3	**1**	**1**		0	0	3.0	**1**	**1**		0	0	10.0	**1**	**1**

The five simulated clinical samples are labeled **A**-**E**. Columns 1: Genomic DNA concentration, ng/μl. Columns 2: Tag4 results. Columns 3: SOLiD results. In columns 2 and 3, "**1**", a majority of the molecular probes for that genome was positive. "0", a majority of the molecular probes for that genome was not positive

Within the Tag4 data, we found one false negative and no false positives. The false negative was for *L. acidophilus *in simulated clinical sample A (SCA). Two of the four *L. acidophilus *molecular probes were positive for SCA. Since 50% is not a majority, we could not call *L. acidophilus *present. None of the four *L. acidophilus *molecular probes was positive for any of the other four simulated clinical samples, not even when two other members of the same genus, *L. brevis *and *L. gasseri*, were present: that is, there was no cross-reaction. For each of the five simulated clinical samples, we counted a large number of bacteria correctly negative: SCA, 34 correct negatives; SCB, 35; SCC, 36; SCD, 35, SCE, 36.

**Figure 2:**
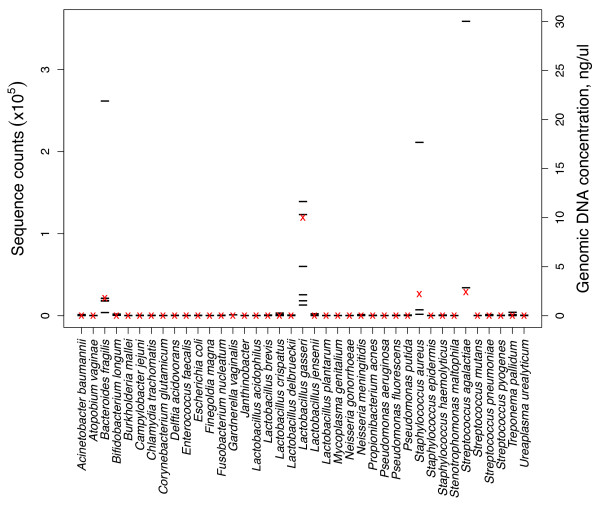
**Quantitative data for the SOLiD assay for simulated clinical sample B (SCB)**. The red crosses indicate the known concentrations of each genomic DNA (right ordinate). The horizontal lines indicate the number of sequence reads for each individual molecular probe (left ordinate). Individual bacteria are listed alphabetically across the abscissa.

Taken as a whole, the results for the simulated clinical samples and the two assays (Tag4 and SOLiD) were in excellent qualitative agreement. However, quantitative agreement between the two methods was not as good. As an example, the SOLiD assay for SCB is shown in Figure 2. (The analogous data for the other four simulated clinical samples are shown in Additional file 1: Figures S1-S4.) The molecular probe leading to the most sequence reads was for *Streptococcus agalactiae *DNA. This number was dramatically different from the number of sequence reads for the second *S. agalactiae *probe (Figure 2). The second highest number of sequence reads was for one molecular probe for *Bacteroides fragilis *DNA. However, *B. fragilis *DNA was present in the least amount of the four genomic DNAs (Figure 2).

### Clinical samples

Our sole criterion for determining which clinical samples (vaginal swabs/DNAs) would be pursued in molecular probe studies was the availability of the DNA after BigDye-terminator (Sanger) sequencing [5]. There was sufficient DNA from twenty-one vaginal swabs to pursue the molecular probe method as assayed on Tag4 arrays. Of these, there were fourteen DNAs sufficient to additionally pursue the molecular probe method as assayed by SOLiD sequencing.

The complete results for all swabs are given in Table S2 (Additional file 1). We present three examples here (Table 2). For clinical sample A08-2, BigDye-terminator sequencing of the 16S ribosomal RNA gene (rDNA) identified two bacteria for which there were molecular probes: *L. crispatus *and *L. jensenii*, in substantially different amounts (Table 2). The same two bacteria were also identified by molecular probe technology as assayed on both Tag4 arrays and by SOLiD sequencing. Based upon the BigDye-terminator data, neither assay produced false negatives or false positives with this clinical sample. (We cannot distinguish the *L. jensenii *probes hybridizing with *L. jensenii *DNA, cross-hybridizing with *L. crispatus *DNA, or both.) Thirty-seven and thirty-eight bacteria were correctly negative with the Tag4 and SOLiD assays, respectively.

**Table 2:** Clinical samples: comparison of BigDye-terminator reads, Tag4 fluorescent signals, and SOLiD reads.

A08-2			
Bacterium	BigDye-terminator reads (%)	Probes/Tag4	Probes/SOLiD

*L. crispatus*	95%	1	1
*L. jensenii*	< 1%	1	1

**A10-4**			

Bacterium	BigDye-terminator reads (%)	Probes/Tag4	Probes/SOLiD

*L. crispatus*	89%	1	1
*L. gasseri*	< 1%	0	0

**A22-3**			

Bacterium	BigDye-terminator reads (%)	Probe/Tag4	Probe/SOLiD

*E. faecalis*		1	0
*L. crispatus*	86%	1	1
*L. jensenii*	13%	1	1
*T. pallidum*		0	1

The BigDye-terminator data are from [5]. For the purposes of this table, those bacteria whose presence was supported by less than ten BigDye-terminator reads have been ignored. Novel bacteria and bacteria without a public genome sequence have also been ignored because they cannot be detected by the molecular probes. "1", a majority of molecular probes for this genome was positive. "0", a majority of molecular probes for this genome was not positive

For clinical sample A10-4 (Table 2), BigDye-terminator sequencing of rDNA identified two bacteria for which there were molecular probes: *L. crispatus *and *L. gasseri*, in substantially different amounts. Both assays detected *L. crispatus*, but neither assay detected *L. gasseri*. Clearly, the *L. gasseri *molecular probes had not cross-reacted with *L. crispatus *DNA. We assume that the amount of *L. gasseri *DNA in clinical sample A10-4 was below the minimum detection limit of the molecular probes, although the minimum detection limit of the molecular probes in clinical samples has not been determined and was probably different for each probe [2]. (The same assumption has been made in an additional six cases: four with the Tag4 assay and two with the SOLiD assay.) Thirty-seven and thirty-eight bacteria were correctly negative with the Tag4 and SOLiD assays, respectively.

Clinical sample A22-3 presents a more complicated picture (Table 2). Both assays correctly identified *L. crispatus *and *L. jensenii *DNAs. However, the Tag4 assay identified *Enterococcus faecalis *DNA, and the SOLiD assay identified *Treponema pallidum *DNA as being present. Nevertheless, thirty-six and thirty-seven bacteria were correctly negative with the Tag4 and SOLiD assays, respectively.

The qualitative agreements between the BigDye-terminator and Tag4 data and the BigDye-terminator and SOLiD data are shown in Table 3. For the twenty-one swabs for which there were Tag4 data, thirteen (62%) were in complete agreement with the BigDye-terminator data. For the fourteen swabs for which there were SOLiD data, 8 (57%) were in complete agreement with the BigDye-terminator data. Five (24%) swabs had apparently false positives by the Tag4 assay and three (21%) by the SOLiD assay. There was no coordination of the apparently false positives between the two assays. As examples, A16-4 had five false positives by the Tag4 assay while the SOLiD assay produced none. A01-1 had four false positives by the SOLiD assay while the Tag4 assay produced none.

**Table 3:** Qualitative agreement of Tag4 and SOLiD assays with BigDye bacteria identifications

ID	BigDye vs. Tag4	BigDye vs. SOLiD
A01-1	A	B
A03-2	A	C
A03-3	C	
A07-1	A	C
A07-2	C	B
A08-2	A	A
A10-2	B	B
A10-4	A	A
A12-2	A	
A13-4	A	
A16-2	A	
A16-3	A	
A16-4	B	A
A17-3	A	A
A19-4	B	A
A20-3	A	A
A22-3	B	B
A23-1	A	
A24-1	C	
A25-2	B	A
A27-2	A	A

A, agreement; B, one (or more) false positive; C, one (or more) false negative; blank: insufficient amount of sample to undertake SOLiD sequencing. In all cases, bacteria inferred to be present, but at a concentration below the minimum detection limit of the molecular probe technology, have been ignored. Only those bacteria for which there were molecular probes were considered

The false negative category was impacted by the undeterminable minimum detection limits for each molecular probe. As an example, for A10-2, the presence of *Corynebacterium glutamicum *was supported by < 1% of the BigDye-terminator reads (Additional file 1: Table S2). Not one of the three *C. glutamicum *molecular probes was positive in either the Tag4 or the SOLiD assay. Leaving aside those seven negatives that are probably explained by the minimum detection limit (Additional file 1: Table S2), there remained five false negatives: 3 (14%) from the Tag4 assay and 2 (14%) from the SOLiD assay. There was no coordination between the two assays. As an example, *L. gasseri *was supported by > 2% of the BigDye-terminator reads for seven swabs. For five of these (A03-2, A07-1, A16-2, A16-3, A17-3), all assays were positive for *L. gasseri *and were in agreement (Additional file 1: Table S2). A07-2 was falsely negative for *L. gasseri *by the Tag4 assay, but correctly positive by the SOLiD assay (Additional file 1: Table S2). In the former case, three of six (not a majority) of the *L. gasseri *molecular probes were positive. For A03-3, none of the six *L. gasseri *molecular probes was positive on the Tag4 array (Additional file 1: Table S2). This last result remains an anomaly. The number of correctly negative bacteria was also important. For the sample with the most apparently false positive Tag4 identifications, A16-4, nevertheless, thirty-one bacteria were correctly negative (Additional file 1: Table S2). For the sample with the most apparently false positive SOLiD identifications, A01-1, nevertheless, thirty-two bacteria were correctly negative (Additional file 1: Table S2).

The large number of SOLiD reads and the high fluorescent intensities on the Tag4 arrays allowed the calculation of Pearson's correlation coefficient between the two assays and between each assay and the number/percent of BigDye-terminator reads. Pearson's correlation coefficient ranges from 1 to -1 and represents a quantitative comparison. The results are shown in Table 4. There were thirteen comparisons of the SOLiD data to the Tag4 data. Eleven (85%) of the coefficients were > 0.5, and nine (69%) of the coefficients were equal to, or greater than, 0.7. There were twelve comparisons of the SOLiD data to the BigDye-terminator data. Seven had a correlation coefficient of 1, and one had a correlation coefficient of 0.84, for a total of 66%. There were seventeen comparisons of the Tag4 data to the BigDye-terminator data. Eleven had a correlation coefficient of 1, and three had a correlation coefficient of > 0.9 for a total of 82%. Thus, overall, the quantitative correlations were excellent.

**Table 4:** Pearson correlation coefficients among the assays

ID	SOLiD *vs*. Tag4	SOLiD *vs*. BigDye	Tag4 *vs*. BigDye
A01-1	0.74	1	1
A03-2	0.45	- 1	1
A03-3			1
A07-1	0.54	- 0.27	- 0.13
A07-2	0.70	- 0.28	- 0.19
A08-2	0.87	1	0.97
A10-2	0.90	1	1
A10-4	0.78	1	1
A13-4			1
A16-2			1
A16-4	0.57		
A17-3	0.46	- 0.13	0.18
A19-4	0.88	1	1
A20-3			1
A22-3	0.76	1	0.95
A23-1			0.97
A25-2	0.83	0.84	1
A27-2	0.88	1	1

## Discussion

Every technology has its advantages and disadvantages. There are two important challenges in detecting bacteria by amplifying and BigDye-terminator (Sanger) sequencing rDNA. (1) rDNA genes are present at multiple copies per genome, and the copy number differs among bacteria [6,7]. (2) The "universal" primers have mismatches to the rDNAs of highly relevant bacteria [8,9]. The negative impact of mismatch between primer and template is substantial [9,10]. Baker *et al. *[11] found that no primer pair had good matches to all bacterial rDNA. Therefore, bacterial genomes with few ribosomal RNA genes and/or with rDNA sequence mismatch to the primers will likely be under-represented in the sequencing library. The same considerations make determining the minimum detection limit problematic. In earlier work, we accomplished extensive modeling of the cost/benefit ratio for BigDye-terminator sequencing [12]. We concluded that four 96-well plates of sequence reads maximized the cost/benefit ratio, which is what we accomplished for these vaginal swabs: 4 × 96 = 384 (minus ~ 5% failed reads = 365 reads). BigDye-terminator sequencing has a very low error rate. Nevertheless, our rule-of-thumb is to require 10 BigDye-terminator reads (~ 3% of the sequence reads) to securely detect a bacterium.

Our molecular probe technology requires a reasonably secure genome sequence for each bacterium and the synthesis of long oligonucleotides. Second generation sequencing is providing bacterial genome sequences faster and cheaper than BigDye-terminator sequencing. The cost of synthesizing oligonucleotides is coming down, while the length is going up.

For the molecular probes, the Homers are based upon single copy sequences. Thus, unlike rDNA-based detection, there is no copy number variation among bacterial genomes that could confound the results. However, to design the Homers, we started with complete genome sequences of specific strains of any given bacterial species. The bacterial genome sequence section of GenBank (presumably) contains only a fraction of the genome sequences of all of the strains for any given species. Thus, a molecular probe may be correctly positive for one strain's genome and correctly negative for another's. This situation would give rise to false negatives in detecting bacteria. We have attempted to minimize this possibility by employing multiple probes per genome and with Homers derived from different parts of the genome sequence.

We have employed two very different assays for the molecular probes: Tag4 array and SOLiD sequencing. There was an apparent lack of good, relative quantitation for both assays, as seen for the simulated clinical samples. With the Tag4 assay, fluorescence intensity is an exponential function of mass and, thereby, inherently difficult to quantitate. However, the assay for each sample requires an individual Tag4 array, and, therefore, each Tag4 assay is independent of the other Tag4 assays. The SOLiD assay requires only counting the number of reads supporting the presence of each bacterium. However, as with any multiplex sequencing, the samples are not independent, as there is a limit to the total number of reads.

Our goal is to produce a technology that will detect bacteria without culture, with commercially available reagents, highly multiplexed, and that will ultimately be fast and inexpensive. Other investigators have invented or adapted technologies toward likely the same goal. Several examples follow. The Insignia system is closest to our technology [13,14]. The system is in two parts. The first part is the publically available software that defines oligonucleotides unique to the target genome of interest [13]. The second part is a quantitative PCR assay (qPCR) [14]. The software is definitely useful. The qPCR assay cannot be multiplexed. Nikolaitchouk *et al. *[15] applied "checkerboard DNA-DNA hybridization" to detect the microbes in the human female genital tract and achieved a 13-plex reaction. Given the complexity of this technology, it is unlikely that very high multiplex can be achieved. DeSantis *et al. *[16] designed and successfully employed a microarray containing 297,851 oligonucleotide probes derived from the rDNA of 842 subfamilies of prokaryotes. Willenbrock *et al. *[17] designed and tested a microarray that contained genome sequences from seven *Escherichia coli *genomes. Their microarray is not commercially available and is unlikely to accommodate very high multiplexing. Dumonceaux *et al. *[18] coupled microbe-specific oligonucleotides to fluorescently labeled microspheres and detected and counted the fluors by flow cytometry, achieving a 9-plex reaction. At present, it is not clear which, if any, of these technologies will turn out to be widely used for detecting bacteria.

While we have concentrated on the detection and identification of bacteria, our molecular probe technology is not limited to that function. Archaea, viruses, even individual genes (such as antibiotic-resistance genes or bacterial toxin genes), could also be detected. The only requirement is sufficient genome sequence to design the unique sequence similarity region of the molecular probe. Because of the multiplex nature of both assays for the molecular probe technology, thousands more probes, representing thousands more entities, may be added at any time [4]. Eventually, the entire human microbiome, in health and in disease, may be assayed in a single reaction tube and employing only commercially available reagents.

## Conclusions

We have presented the first use of our molecular probe technology to detect bacteria in clinical samples. In addition to the Tag4 array assay, we introduced a second assay employing SOLiD sequencing. The SOLiD sequencing assay allowed the processed samples to be combined before sequencing for even greater multiplexing. The correlations among those two assays and the previously published BigDye-terminator sequencing assay were excellent.

## Methods

### Human subjects

We have published the relevant information concerning the patients who were recruited and consented for this study [5]. All patients were enrolled at the University of California, San Francisco (U.C.S.F). This protocol was approved by the Committee on Human Research at U.C.S.F and by the Committee on the Use of Human Subjects in Research at Stanford University.

### Total DNA from vaginal swabs

Swabs of the posterior vaginal fornix were taken at U.C.S.F., as described [12]. The frozen, de-identified vaginal swabs were transferred to the Stanford Genome Technology Center (S.G.T.C.). We purified total DNA from each vaginal swab employing a Qiagen DNeasy Blood and Tissue Kit. The final step was dialysis and concentration with Amicon Ultra Centrifugal Filters (0.5 ml, 100 K). Each total DNA preparation for each swab was frozen at-70°C in two ~10 μl aliquots until use.

### BigDye-terminator sequencing to detect the bacteria present on the swabs

We have published our procedures for PCR amplifying and cloning the rDNA genes, and sequencing the rDNA genes employing BigDye-terminator chemistry (Sanger sequencing) [12]. In brief, we achieved four 96-well plates of sequence reads per swab [5]. We assembled the individual sequence reads into contigs employing the KB Basecaller [19]. Importantly, we hand edited the contigs. We compared the consensus sequence of each contig to the data in the Ribosomal Database Project [RDP; [20]. Technically, the annealing of a molecular probe to a template only confirmed the presence of a particular sequence. We inferred the presence of a particular bacterium from the similarity of any given contig consensus sequence to its closest match in the RDP.

### Molecular probes

We have published the detailed design of our molecular probes [2]. In brief, there are three domains within the molecular probes (Figure 1a). The first domain is a contiguous 40-base sequence (the "Homer"), divided into two 20-mers, unique to the genome of the target bacteria. A list of the bacteria and their corresponding genome sequences is provided in (Additional file 1: Table S3) [21]. The second domain is a twenty base oligonucleotide barcode from the Affymetrix Tag4 array [22]. The third domain is a 36-base universal PCR amplification sequence [23]. Thus, the molecular probes are 96 bases in length. We purchased the probes as 5'-phosphorylated and PAGE-purified from Integrated DNA Technologies. The molecular probe mixture contained 192 molecular probes representing 40 bacteria [2]. There was an average of (192/40 =) 4.8 molecular probes per bacterial genome with a range of 2-to-7.

Our procedure is to anneal the molecular probes to the denatured DNA target. Where there is sufficient sequence similarity between probe and target, a circular DNA forms (Figure 1b). No bases are missing. Only a phosphodiester bond is missing between the 5' and 3' bases of the probe. Enzymatic ligation produces single-stranded circular DNA. Exonuclease digestion removes all linear DNA. PCR primers based upon the 36-base universal amplification sequence are employed to PCR amplify the circular DNA.

For the purposes of this work, we excluded from the analysis those bacteria with insufficient public genome sequence to design molecular probes. This category included novel bacteria, which were defined as previously [12]. The novel rDNA sequences have been deposited in GenBank: accession numbers [HQ293151-HQ293203].

### Assaying the molecular probes on Tag4 arrays

The Tag4 array contains 8-μm features. Each 20-mer barcode is replicated and dispersed five times on the array [22]. We have published the detailed procedures for assaying the molecular probes on the Tag4 array [2]. In all cases, the final read-out was fluorescence intensity.

On all the Tag4 arrays, the six molecular probes for *L. delbrueckii *produced no signals above background (unoccupied 20-mers on the Tag4 array). Therefore, we employed these six probes as the negative controls. We calculated the average fluorescence signal and standard deviation for the six *L. delbrueckii *probes. To minimize false positives at this stage of the development of the molecular probe technology, we calculated the average plus five standard deviations. We employed that number as the cut-off between negative and positive for each molecular probe on a Tag4 array. Also to minimize false positives at this stage of the development of the molecular probe technology, we required concordance of the data. A majority (> 50%) of the molecular probes for any given bacterium must have been positive for us to call a bacterium present. There is a potential problem with this procedure that is related to possible strain variation in genome sequence: *i.e*., genome sequence variation within the same species. Any given molecular probe could be authentically positive for one strain and authentically negative for another.

For the five simulated clinical samples, five molecular probes were positive for all samples whether their corresponding DNA was present or not: one probe each for *Acinetobacter baumannii *(ED211; leaving four probes), *B. fragilis *(ED141; leaving four probes), *Bifidobacterium longum *(ED611; leaving four probes), and two probes for *T. pallidum *(ED317 and ED322; leaving three probes). Therefore, the data from these five molecular probes were excluded from the analyses. Two of three probes for *Gardnerella vaginalis *(ED116 and ED121B) were also positive for all five simulated clinical samples, when there was no *G. vaginalis *DNA present in any sample. Since we would not call a bacterium present or absent on the basis of one molecular probe, *G. vaginalis *was excluded from the analyses. What remained for evaluation of the simulated clinical samples were 183 molecular probes representing 39 bacteria.

We conducted an analogous process for detecting promiscuous molecular probes within the Tag4 data for the twenty-one clinical samples. Again, to minimize false positives at this stage of the development of the molecular probe technology, we identified molecular probes positive for ten or more (equal to, or greater than, 50%) of the clinical samples (excluding *Lactobacillus *probes). We abandoned the data therefrom: two probes for *A. baumannii *(ED212 and ED213; leaving three probes) were positive for twenty and nineteen samples, respectively; two probes for *G. vaginalis *(ED116 and ED121B; leaving one probe); two probes for *Streptococcus pneumoniae *(ED276 and ED277; leaving three probes) were positive for twelve and thirteen samples, respectively; one probe for *S. pyogenes *(ED413; leaving three probes) was positive for ten samples; and one probe for *Fusobacterium nucleatum *(ED559; leaving five probes) was positive for seventeen samples. The data from all six *Enterobacter *probes (leaving none) were excluded. *G. vaginalis *and *Pseudomonas aeruginosa *were left with only one molecular probe each. Since we would not make a present/absent determination on the basis of one molecular probe, *G. vaginalis and P. aeruginosa *were removed from consideration within the clinical samples. Only two promiscuous probes were shared between both sets of Tag4 data: ED116 and ED121B (*G. vaginalis*). Whereas one *A. baumannii *probe (ED211) was promiscuous in the simulated clinical sample data, two other *A. baumannii *probes (ED212 and ED213) were promiscuous in the clinical sample data. What remained for the authentic clinical samples assayed on the Tag4 array were (192 - 17 =) 175 molecular probes representing 37 bacteria.

Public genome sequence for *L. crispatus *and *L. jensenii *appeared only after the design of all of the other molecular probes [2]. These two genome sequences were derived from short shotgun pyrosequencing reads, which had been assembled into dozens of contigs for each genome. Thus, these two genome sequences were far from ideal for the purpose of designing unique 40-mer Homers. Nevertheless, given the importance of *L. crispatus *and *L. jensenii *to the health of the human vagina, we designed molecular probes for these two bacterial DNAs. Presumably, as a direct consequence of the incompleteness of the two genome sequences, the molecular probes for *L. crispatus *and *L. jensenii *cross-reacted with each other's DNA and sometimes with *L. brevis *and *L. gasseri *DNAs as well. In addition, although the sequences for the existing molecular probes for *L. brevis *and *L. gasseri *were compared to the *L. crispatus *and *L. jensenii *genome sequences with only negative results, the *L. brevis *and *L. gasseri *probes sometimes reacted with *L. crispatus *and *L. jensenii *DNAs in the clinical samples. To avoid confusion, only those *Lactobacillus *species identified by BigDye-terminator sequencing appear in the tables. The probes for *L. acidophilus, L. delbrueckii*, and *L. plantarum *did not cross-react with other *Lactobacillus *DNAs.

The microarray data are MIAME compliant and have been deposited in the Array Express website: accession: [E-MEXP-2958]. The CEL (cell intensity) files of the microarray data are publicly available on the Stanford Genome Technology Center website http://med.stanford.edu/sgtc/research/download/.

### Assaying the molecular probes by Sequencing by Oligonucleotide Ligation and Detection (SOLiD)

The primers used to amplify the product for SOLiD sequencing are presented in Table S1 (Additional file 1). The primer sequences were based upon published designs [24].

SOLiD sequencing, a sequencing-by-ligation technology (Applied Biosystems, Foster City, CA), was performed at the University of California Santa Cruz Genome Sequencing Center. We have published our procedure for the library preparation of the samples for SOLiD sequencing [25,26]. We followed the manufacturer's protocols for the barcoded SOLiD System 3.0 Fragment Library. We prepared the samples according to the manufacturer's protocols for the emulsion PCR step of SOLiD sequencing. We processed the samples with the SOLiD Version 3.0 system, producing 50 bases of sequencing information for each read.

Molecular tag sequences were ligated and amplified *in silico *to create the expected reference sequences. The forward and reverse complements of all molecular tag reference sequences were translated from base space into color space using a custom perl script. We trimmed 20 bases from the 5' end of each read to remove the adapter.

We aligned the sequence reads to each reference molecular tag sequence using a publically available Smith-Waterman local alignment in colorspace with affine gap penalties [27]. We determined an alignment threshold corresponding to an alpha value of 0.05 by aligning 10 million random reads to each reference sequence. For each read, we kept the reference sequence with the highest scoring alignment if its score exceeded the empirically derived threshold. The final read-out was the number of reads corresponding to each molecular probe.

Analogously to the processing of the Tag4 data, we employed the data for the six probes for *L. delbrueckii *as the negative control. The average number of SOLiD reads and standard deviation for the six were calculated. Again, to minimize false positives at this stage of the development of the molecular probe technology, we used the average plus five standard deviations as the cut-off between negative and positive for each molecular probe. Also to minimize the number of false positives at this stage of the development of the molecular probe technology, concordance of the data was required. A majority of the molecular probes for any given microbe must have been positive to score the microbe as present. The same caveats as for the Tag4 data analysis apply.

We identified promiscuous molecular probes for the five simulated clinical samples. ED116 (*G. vaginalis*) and ED675 (*L. jensenii) *were positive for all five simulated clinical samples, when neither DNA was present in any. ED611 (*B. longum*) and ED121B (*G. vaginalis*) were positive for four of the five simulated clinical samples. Therefore, the data from these four probes were excluded from the analyses. As only one *G. vaginalis *probe remained, *G. vaginalis *was removed from further consideration. That left 187 molecular probes representing 39 bacteria.

There were SOLiD data for fourteen clinical samples. Since these were sequenced with the simulated clinical samples, the identical negative control was employed. We identified promiscuous molecular probes for the clinical samples. We excluded the data for any probe positive for seven (50%) or more samples (except *Lactobacillus*). That group included sixteen molecular probes: *A. baumannii *(ED211, 13/14; ED212, 7/14; ED213, 8/14; leaving two probes), *B. fragilis *(ED141, 12/14; leaving four probes), *B. longum *(ED611, 13/14; ED614, 12/14; ED619, 7/14; leaving two probes), *G. vaginalis *(ED116, 13/14; ED119, 10/14; ED121B, 14/14; leaving no probes), *L. jensenii *(ED675, 14/14; leaving five probes), *Staphylococcus aureus *(ED236, 12/14; leaving two probes), *S. agalactiae *(ED263, 12/14; leaving one probe), *T. pallidum *(ED317, 14/14; ED322, 9/14; leaving three probes), and *Ureaplasma urealyticum *(ED640, 12/14; leaving four probes). Unfortunately, there were no remaining molecular probes for *G. vaginalis*, and *S. agalactiae *was left with only one molecular probe. Since we would not make a present/absent determination on the basis of one molecular probe, *S. agalactiae *was removed from consideration within the clinical samples. (Interestingly, the one remaining *S. agalactiae *molecular probe, ED265, was never positive for any sample.) What remained for the authentic clinical samples were (192 - 17 =) 175 molecular probes representing 38 bacteria.

The four promiscuous probes from the SOLiD data for the simulated clinical samples were also promiscuous within the clinical samples: ED116 and ED121B (*G. vaginalis*), ED611 (*B. longum*), and ED675 (*L. jensenii*). Overall, only two probes were promiscuous in all four sets of data: ED116 and ED121B (*G. vaginalis*). ED611 (*B. longum*) was promiscuous in three of the four sets. No other probes were that promiscuous.

### Correlations

Bacterial species identified by BigDye-terminator sequencing and by molecular barcodes were used to investigate correlations among the two methods and three assays. Raw CEL files were obtained for each Tag4 assay. The fluorescent intensity was calculated for each molecular barcode. The number of reads from SOLiD sequencing was counted for each barcode. We calculated Pearson's correlation coefficient for samples assessed by both SOLiD sequencing and Tag4 arrays. For the "cut-off" method, we preserved the number of counts for each probe only if that number exceeded the number of counts for the negative control molecular probes. For swabs A12-2, A16-3, and A24-1, less than one bacterium was identified. Therefore, we could not calculate the correlation coefficients for these three samples.

## Abbreviations

Homer: the molecular probe's 40 base homology region; MIP: molecular inversion probe; qPCR: quantitative PCR; RDP: Ribosomal Database Project; rDNA: 16S ribosomal RNA gene; S.G.T.C.: Stanford Genome Technology Center; SOLiD: Sequencing by Oligonucleotide Ligation and Detection; SCA: simulated clinical sample A *etc*. for B, C, D, and E; Tag4: Affymetrix GenFlex Tag16K Array v2 array; U.C.S.F.: University of California San Francisco.

## Authors' contributions

RWH and RPStO designed the experiments. MF carried out the sequencing reactions, processed and assembled the sequence reads, and compared the consensus sequences to the data in the RDP. MF and RWH hand edited the contigs. RWH performed the first steps in both of the molecular probe procedures and wrote this manuscript. MM and AMA performed the Tag4 microarray assays. RPStO and RWH analyzed the Tag4 microarray data. HK and NP performed the SOLiD assays and analyzed the data. HK performed the statistical analyses of the data. JST validated the statistical analyses. LCG provided the vaginal swabs. RWD provided the intellectual, physical, and financial milieu for these experiments. All authors read and approved the final manuscript.

## Author information

Ronald W. Davis is a co-holder of the patent for molecular inversion probes.

## Supplementary Material

Additional file 1 **Table S1**. Amplification primers for subsequent SOLiD sequencing. **Table S2**. Clinical samples: comparison of BigDye-terminator reads, Tag4 fluorescent signals, and SOLiD reads. The BigDye-terminator data are from [5]. **Table S3**. Bacteria and the RefSeq numbers for their genome sequences. **Figure S1**. Quantitative data for the SOLiD assay for simulated clinical sample A (SCA). **Figure S2**. Quantitative data for the SOLiD assay for simulated clinical sample C (SCC). **Figure S3**. Quantitative data for the SOLiD assay for simulated clinical sample D (SCD). **Figure S4**. Quantitative data for the SOLiD assay for simulated clinical sample E (SCE). Click here for file
