# Supplementary material for: Molecular probe technology detects bacteria without culture
Source: BMC Microbiol. 2012 Mar 9;12:29. doi: 10.1186/1471-2180-12-29 (PMC3316761; doi:10.1186/1471-2180-12-29)

**Additional files**

**Tables**

**Additional file 1: Table S1.** Amplification primers for subsequent SOLiD sequencing. The letters in bold are the 10-mer indexing barcodes. Primer sequences were derived from [Smith *et al*., 2010]. Barcodes are in bold.

**Universal forward primer.**

5'-CCTCTCTATGGGCAGTCGGTGATTACTGAGGTCGGTACACTCT-3'

**Individual reverse primers.**

Simulated clinical sample A: 5'-CTGCCCCGGGTTCCTCATTCTCT**AGGCTGTCTA**CTGCTGTACGGCCAAGGCGAGTAGCCGTGACTATCGACT-3'

Simulated clinical sample B: 5'-CTGCCCCGGGTTCCTCATTCTCT**GTGACCTACT**CTGCTGTACGGCCAAGGCGAGTAGCCGTGACTATCGACT-3'

Simulated clinical sample C: 5'-CTGCCCCGGGTTCCTCATTCTCT**GCGTATTGGG**CTGCTGTACGGCCAAGGCGAGTAGCCGTGACTATCGACT-3'

Simulated clinical sample D: 5'-CTGCCCCGGGTTCCTCATTCTCT**AAGGGATTAC**CTGCTGTACGGCCAAGGCGAGTAGCCGTGACTATCGACT-3'

Simulated clinical sample E: 5'-CTGCCCCGGGTTCCTCATTCTCT**GTTACGATGC**CTGCTGTACGGCCAAGGCGAGTAGCCGTGACTATCGACT-3'

Clinical sample A08-2: 5'-CTGCCCCGGGTTCCTCATTCTCT**ATGGGTGTTT**CTGCTGTACGGCCAAGGCGAGTAGCCGTGACTATCGACT-3'

Clinical sample A10-4: 5'-CTGCCCCGGGTTCCTCATTCTCT**GAGTCCGGCA**CTGCTGTACGGCCAAGGCGAGTAGCCGTGACTATCGACT-3'

Clinical sample A19-4: 5'-CTGCCCCGGGTTCCTCATTCTCT**AATCGAAGAG**CTGCTGTACGGCCAAGGCGAGTAGCCGTGACTATCGACT-3'

Clinical sample A01-1: 5'-CTGCCCCGGGTTCCTCATTCTCT**GGTCGTCGAA**CTGCTGTACGGCCAAGGCGAGTAGCCGTGACTATCGACT-3'

Clinical sample A22-3: 5'-CTGCCCCGGGTTCCTCATTCTCT**GAGGGATGGC**CTGCTGTACGGCCAAGGCGAGTAGCCGTGACTATCGACT-3'

Clinical sample A10-2: 5'-CTGCCCCGGGTTCCTCATTCTCT**GAAGGCTTGC**CTGCTGTACGGCCAAGGCGAGTAGCCGTGACTATCGACT-3'

Clinical sample A17-3: 5'-CTGCCCCGGGTTCCTCATTCTCT**GTAATTGTAA**CTGCTGTACGGCCAAGGCGAGTAGCCGTGACTATCGACT-3'

Clinical sample A16-4: 5'-CTGCCCCGGGTTCCTCATTCTCT**GTCATCAAGT**CTGCTGTACGGCCAAGGCGAGTAGCCGTGACTATCGACT-3'

Clinical sample A03-2: 5'-CTGCCCCGGGTTCCTCATTCTCT**GCATGTCACC**CTGCTGTACGGCCAAGGCGAGTAGCCGTGACTATCGACT-3'

Clinical sample A27-2: 5'-CTGCCCCGGGTTCCTCATTCTCT**CTAGTAAGAA**CTGCTGTACGGCCAAGGCGAGTAGCCGTGACTATCGACT-3'

Clinical sample A07-1: 5'-CTGCCCCGGGTTCCTCATTCTCT**TAAAGTGGCG**CTGCTGTACGGCCAAGGCGAGTAGCCGTGACTATCGACT-3'

Clinical sample A07-2: 5'-CTGCCCCGGGTTCCTCATTCTCT**AAGTAATGTC**CTGCTGTACGGCCAAGGCGAGTAGCCGTGACTATCGACT-3'

Clinical sample A20-3: 5'-CTGCCCCGGGTTCCTCATTCTCT**ATGTCATAAG**CTGCTGTACGGCCAAGGCGAGTAGCCGTGACTATCGACT-3'

Clinical sample A25-2: 5'-CTGCCCCGGGTTCCTCATTCTCT**AAGCAGGAGT**CTGCTGTACGGCCAAGGCGAGTAGCCGTGACTATCGACT-3'

**Additional file 2: Table S2.** Clinical samples: comparison of BigDye-terminator reads, Tag4 fluorescent signals, and SOLiD reads. The BigDye-terminator data are from [Hyman *et al*., 2012]. For the purposes of this table, those bacteria whose presence was supported by less than ten BigDye-terminator reads have been ignored. Novel bacteria and bacteria without a public genome sequence have also been ignored because they cannot be detected by the molecular probes. “1”, a majority of molecular probes for this genome was positive. “0”, a majority of molecular probes for this genome was not positive.

**A01-1**

| Bacterium | BigDye-terminator reads (%) | Probe/Tag4 | Probe/SOLiD |
| --- | --- | --- | --- |
| *B. longum* |  | 0 | 1 |
| *C. jejuni* |  | 0 | 1 |
| *L. crispatus* | 96% | 1 | 1 |
| *L. jensenii* | < 1% | 1 | 1 |
| *P. aeruginosa* |  | 0 | 1 |
| *T. pallidum* |  | 0 | 1 |

**A03-2**

| Bacterium | BigDye-terminator reads (%) | Probe/Tag4 | Probe/SOLiD |
| --- | --- | --- | --- |
| *L. gasseri* | 44% | 1 | 1 |
| *L. jensenii* | 44% | 1 | 0 |

**A03-3**

| Bacterium | BigDye-terminator reads (%) | Probe/Tag4 |
| --- | --- | --- |
| *L. gasseri* | 88% | 0 |

**A07-1**

| Bacterium | BigDye-terminator reads (%) | Probes/Tag4 | Probes/SOLiD |
| --- | --- | --- | --- |
| *L. gasseri* | 16% | 1 | 1 |
| *L. jensenii* | 72% | 1 | 0 |

**A07-2**

| Bacterium | BigDye-terminator reads (%) | Probes/Tag4 | Probes/SOLiD |
| --- | --- | --- | --- |
| *E. coli* |  | 0 | 1 |
| *L. gasseri* | 21% | 0 | 1 |
| *L. jensenii* | 75% | 1 | 1 |

**A08-2**

| Bacterium | BigDye-terminator reads (%) | Probes/Tag4 | Probes/SOLiD |
| --- | --- | --- | --- |
| *L. crispatus* | 95% | 1 | 1 |
| *L. jensenii* | < 1% | 1 | 1 |

**A10-2**

| Bacterium | BigDye-terminator reads (%) | Probes/Tag4 | Probes/SOLiD |
| --- | --- | --- | --- |
| *B. fragilis* |  | 1 | 0 |
| *C. glutamicum* | < 1% | 0 | 0 |
| *L. crispatus* | 95% | 1 | 1 |
| *L. gasseri* | 1% | 0 | 1 |
| *S. pyogenes* |  | 1 | 0 |
| *T. pallidum* |  | 0 | 1 |

**A10-4**

| Bacterium | BigDye-terminator reads (%) | Probes/Tag4 | Probes/SOLiD |
| --- | --- | --- | --- |
| *L. crispatus* | 89% | 1 | 1 |
| *L. gasseri* | < 1% | 0 | 0 |

**A12-2**

| Bacterium | BigDye-terminator reads (%) | Probes/Tag4 |
| --- | --- | --- |
| *L. jensenii* | 74% | 1 |

**A13-4**

| Bacterium | BigDye-terminator reads | Probes/Tag4 |
| --- | --- | --- |
| *L. jensenii* | 88% | 1 |

**A16-2**

| Bacterium | BigDye-terminator reads (%) | Probes/Tag4 |
| --- | --- | --- |
| *L. gasseri* | 100% | 1 |

**A16-3**

| Bacterium | BigDye-terminator reads (%) | Probes/Tag4 |
| --- | --- | --- |
| *L. gasseri* | 100% | 1 |

**A16-4**

| Bacterium | BigDye-terminator reads (%) | Probes/Tag4 | Probes/SOLiD |
| --- | --- | --- | --- |
| *B. longum* |  | 1 | 0 |
| *Janthinobacter* |  | 1 | 0 |
| *L. crispatus* | 100% | 1 | 1 |
| *N. gonorrhoeae* |  | 1 | 0 |
| *S. pyogenes* |  | 1 | 0 |
| *U. urealyticum* |  | 1 | 0 |

**A17-3**

| Bacterium | BigDye-terminator reads (%) | Probes/Tag4 | Probes/SOLiD |
| --- | --- | --- | --- |
| *L. gasseri* | 70% | 1 | 1 |

**A19-4**

| Bacterium | BigDye-terminator reads (%) | Probes/Tag4 | Probes/SOLiD |
| --- | --- | --- | --- |
| *L. crispatus* | 97% | 1 | 1 |
| *L. gasseri* | 2% | 0 | 0 |
| *M. genitalium* |  | 1 | 0 |

**A20-3**

| Bacterium | BigDye-terminator reads (%) | Probe/Tag4 | Probe/SOLiD |
| --- | --- | --- | --- |
| *L. crispatus* | 81% | 1 | 1 |

**A22-3**

| Bacterium | BigDye-terminator reads (%) | Probe/Tag4 | Probe/SOLiD |
| --- | --- | --- | --- |
| *E. faecalis* |  | 1 | 0 |
| *L. crispatus* | 86% | 1 | 1 |
| *L. jensenii* | 13% | 1 | 1 |
| *T. pallidum* |  | 0 | 1 |

**A23-1**

| Bacterium | BigDye-terminator reads (%) | Probe/Tag4 |
| --- | --- | --- |
| *L. crispatus* | 96% | 1 |
| *L. jensenii* | 2% | 1 |
| *L. gasseri* | 2% | 0 |

**A24-1**

| Bacterium | BigDye-terminator reads (%) | Probe/Tag4 |
| --- | --- | --- |
| *L. jensenii* | 93% | 0 |

**A25-2**

| Bacterium | BigDye-terminator reads (%) | Probe/Tag4 | Probe/SOLiD |
| --- | --- | --- | --- |
| *A. baumannii* |  | 1 | 0 |
| *L. crispatus* | 86% | 1 | 1 |
| *L. jensenii* | < 1% | 1 | 1 |
| *M. genitalium* |  | 1 | 0 |

**A27-2**

| Bacterium | BigDye-terminator reads (%) | Probe/Tag4 | Probe/SOLiD |
| --- | --- | --- | --- |
| *L. crispatus* | 95% | 1 | 1 |
| *L. jensenii* | 4% | 1 | 1 |

**Additional file 3: Table S3.** Bacteria and RefSeq genome sequence numbers.

| nc_id | genus | species | strain | comments | comments |
| --- | --- | --- | --- | --- | --- |
| NC_008752 | Acidovorax | avenae | subsp__citrulli_AAC00_1 |  |  |
| NC_008782 | Acidovorax | sp. | JS42 |  |  |
| NC_009085 | Acinetobacter | baumannii | ATCC_17978 |  |  |
| NC_010410 | Acinetobacter | baumannii | AYE |  |  |
| NC_010400 | Acinetobacter | baumannii, | Complete genome |  |  |
| NC_005966 | Acinetobacter | sp. | ADP1 |  |  |
| NC_008570 | Aeromonas | hydrophila | subsp__hydrophila_ATCC_7966 |  |  |
| NC_003228 | Bacteroides | fragilis | NCTC_9343 |  |  |
| NC_006347 | Bacteroides | fragilis | YCH46 |  |  |
| NC_004663 | Bacteroides | thetaiotaomicron | VPI_5482 |  |  |
| NC_009614 | Bacteroides | vulgatus | ATCC_8482 |  |  |
| NC_008618 | Bifidobacterium | adolescentis | ATCC_15703 |  |  |
| NC_004307 | Bifidobacterium | longum | NCC2705 |  |  |
| NC_010816 | Bifidobacterium | longum | DJO10A |  |  |
| NC_010551 | Burkholderia | ambifaria | MC40_6_chromosome_1 |  |  |
| NC_008060 | Burkholderia | cenocepacia | AU_1054_chromosome_1 |  |  |
| NC_010508 | Burkholderia | cenocepacia | MC0_3_chromosome_1 |  |  |
| NC_008390 | Burkholderia | cepacia | AMMD_chromosome_1 |  |  |
| NC_008836 | Burkholderia | mallei | NCTC_10229_chromosome_I |  |  |
| NC_010084 | Burkholderia | multivorans | ATCC_17616_chromosome_1 |  |  |
| NC_006350 | Burkholderia | pseudomallei | K96243_chromosome_1 |  |  |
| NC_009076 | Burkholderia | pseudomallei | 1106a_chromosome_I |  |  |
| NC_007651 | Burkholderia | thailandensis | E264_chromosome_I |  |  |
| NC_009256 | Burkholderia | vietnamiensis | G4_chromosome_1 |  |  |
| NC_007951 | Burkholderia | xenovorans | LB400_chromosome_1 |  |  |
| NC_009714 | Campylobacter | hominis | ATCC_BAA_381 |  |  |
| NC_002163 | Campylobacter | jejuni | subsp__jejuni_NCTC_11168 |  |  |
| NC_003912 | Campylobacter | jejuni | RM1221 |  |  |
| NC_009707 | Campylobacter | jejuni | subsp__doylei_269_97 |  |  |
| NC_009839 | Campylobacter | jejuni | subsp__jejuni_81116 |  |  |
| NC_000117 | Chlamydia | trachomatis | D_UW_3_CX |  |  |
| NC_007429 | Chlamydia | trachomatis | A_HAR_13 |  |  |
| NC_010280 | Chlamydia | trachomatis | L2b_UCH_1_proctitis |  |  |
| NC_010287 | Chlamydia | trachomatis | 434_Bu |  |  |
| NC_008261 | Clostridium | perfringens | ATCC_13124 |  |  |
| NC_008262 | Clostridium | perfringens | SM101 |  |  |
| NC_003450 | Corynebacterium | glutamicum | ATCC_13032 |  |  |
| NC_006958 | Corynebacterium | glutamicum | ATCC_13032 |  |  |
| NC_009342 | Corynebacterium | glutamicum | R |  |  |
| NC_010002 | Delftia | acidovorans | SPH_1 |  |  |
| NC_009778 | Enterobacter | sakazakii | ATCC_BAA_894 |  |  |
| NC_009436 | Enterobacter | sp. | "638" |  |  |
| NC_004668 | Enterococcus | faecalis | V583 |  |  |
| AC_000091 | Escherichia | coli | W3110_DNA |  |  |
| NC_000913 | Escherichia | coli | str__K_12_substr__MG1655 |  |  |
| NC_004431 | Escherichia | coli | CFT073 |  |  |
| NC_007946 | Escherichia | coli | UTI89 |  |  |
| NC_008253 | Escherichia | coli | "536" |  |  |
| NC_008563 | Escherichia | coli | APEC_O1 |  |  |
| NC_009800 | Escherichia | coli | HS |  |  |
| NC_010473 | Escherichia | coli | str__K_12_substr__DH10B |  |  |
| NC_010376 | Finegoldia | magna | ATCC_29328 |  |  |
| NC_009441 | Flavobacterium | johnsoniae | UW101 |  |  |
| NC_009613 | Flavobacterium | psychrophilum | JIP02_86 |  |  |
| NC_003454 | Fusobacterium | nucleatum | subsp__nucleatum_ATCC_25586 |  |  |
| NC_009659 | Janthinobacterium | sp. | Marseille |  |  |
| NC_006814 | Lactobacillus | acidophilus | NCFM |  |  |
| NC_008497 | Lactobacillus | brevis | ATCC_367 |  |  |
| NC_008054 | Lactobacillus | delbrueckii | subsp__bulgaricus_ATCC_11842 |  |  |
| NC_008529 | Lactobacillus | delbrueckii | subsp__bulgaricus_ATCC_BAA_365 |  |  |
| NC_008530 | Lactobacillus | gasseri | ATCC_33323 |  |  |
| NC_004567 | Lactobacillus | plantarum | WCFS1 |  |  |
| NC_000908 | Mycoplasma | genitalium | G37 |  |  |
| NC_002946 | Neisseria | gonorrhoeae | FA_1090 |  |  |
| NC_003112 | Neisseria | meningitidis | MC58 |  |  |
| NC_003116 | Neisseria | meningitidis | Z2491 |  |  |
| NC_008767 | Neisseria | meningitidis | FAM18 |  |  |
| NC_010120 | Neisseria | meningitidis | "053442" |  |  |
| NC_006085 | Propionibacterium | acnes | KPA171202 |  |  |
| NC_002516 | Pseudomonas | aeruginosa | PAO1 |  |  |
| NC_008463 | Pseudomonas | aeruginosa | UCBPP_PA14 |  |  |
| NC_009656 | Pseudomonas | aeruginosa | PA7 |  |  |
| NC_008027 | Pseudomonas | entomophila | L48 |  |  |
| NC_004129 | Pseudomonas | fluorescens | Pf_5 |  |  |
| NC_007492 | Pseudomonas | fluorescens | PfO_1 |  |  |
| NC_009439 | Pseudomonas | mendocina | ymp |  |  |
| NC_002947 | Pseudomonas | putida | KT2440 |  |  |
| NC_002947 | Pseudomonas | putida | KT2440 |  |  |
| NC_009512 | Pseudomonas | putida | F1 |  |  |
| NC_010322 | Pseudomonas | putida | GB_1 |  |  |
| NC_010501 | Pseudomonas | putida | W619 |  |  |
| NC_009434 | Pseudomonas | stutzeri | A1501 |  |  |
| NC_002758 | Staphylococcus | aureus | subsp__aureus_Mu50 |  |  |
| NC_002951 | Staphylococcus | aureus | subsp__aureus_COL |  |  |
| NC_003923 | Staphylococcus | aureus | subsp__aureus_MW2 |  |  |
| NC_009487 | Staphylococcus | aureus | subsp__aureus_JH9 |  |  |
| NC_009632 | Staphylococcus | aureus | subsp__aureus_JH1 |  |  |
| NC_009641 | Staphylococcus | aureus | subsp__aureus_str__Newman |  |  |
| NC_009782 | Staphylococcus | aureus | subsp__aureus_Mu3 |  |  |
| NC_002976 | Staphylococcus | epidermidis | RP62A |  |  |
| NC_004461 | Staphylococcus | epidermidis | ATCC_12228 |  |  |
| NC_010943 | Stenotrophomonas | maltophilia | K279a |  |  |
| NC_004116 | Streptococcus | agalactiae | 2603V_R |  |  |
| NC_004368 | Streptococcus | agalactiae | NEM316 |  |  |
| NC_007432 | Streptococcus | agalactiae | A909 |  |  |
| NC_004350 | Streptococcus | mutans | UA159 |  |  |
| NC_004606 | Streptococcus | pyogenes | SSI_1 |  |  |
| NC_006086 | Streptococcus | pyogenes | MGAS10394 |  |  |
| NC_008023 | Streptococcus | pyogenes | MGAS2096 |  |  |
| NC_009332 | Streptococcus | pyogenes | str__Manfredo |  |  |
| NC_000919 | Treponema | pallidum | subsp__pallidum_str__Nichols |  |  |
| NC_011374 | Ureaplasma | urealyticum | serovar_10_str_ATCC_33699 | species file empty rerun 2/9/09 | still empty only a few genus matches |
|  |  |  |  |  |  |
|  |  |  |  |  |  |
|  |  |  |  |  |  |
|  |  |  |  |  |  |
|  |  |  |  |  |  |

**Figures.**

**Additional file 4: Figure S1.** Quantitative data for the SOLiD assay for simulated clinical sample A (SCA). The red crosses indicate the known concentrations of each genomic DNA (right ordinate). The horizontal lines indicate the number of sequence reads for each individual molecular probe (left ordinate). Individual bacteria are listed alphabetically across the abscissa. The number of reads for *L. acidophilus* is just above background.


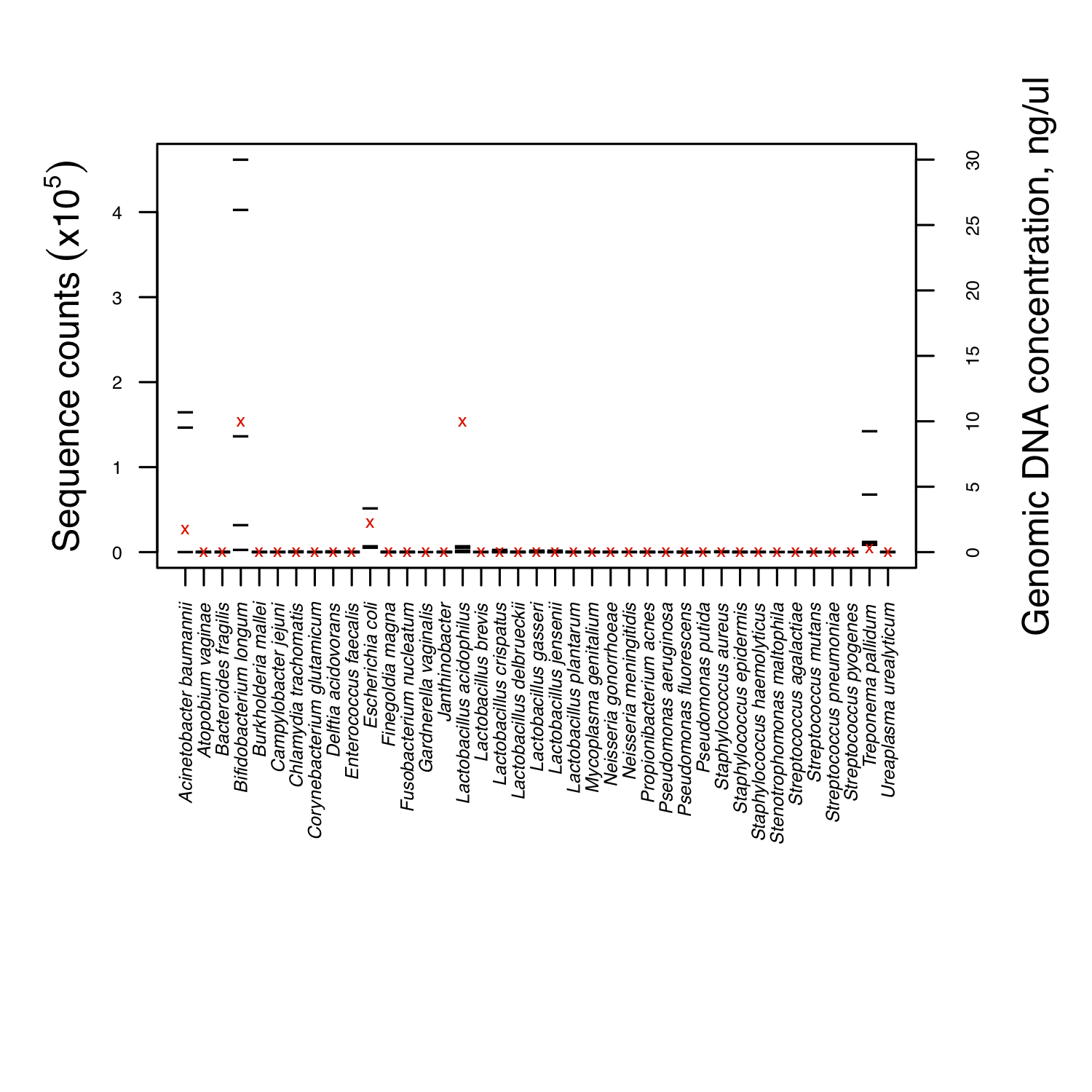
 **Additional file 5: Figure S2.** Quantitative data for the SOLiD assay for simulated clinical sample C (SCC). The red crosses indicate the known concentrations of each genomic DNA (right ordinate). The horizontal lines indicate the number of sequence reads for each individual molecular probe (left ordinate). Individual bacteria are listed alphabetically across the abscissa. Some of the molecular probes for *L. crispatus* and *L. jensenii* DNAs registered positive by cross-reaction with *L. gasseri* DNA.


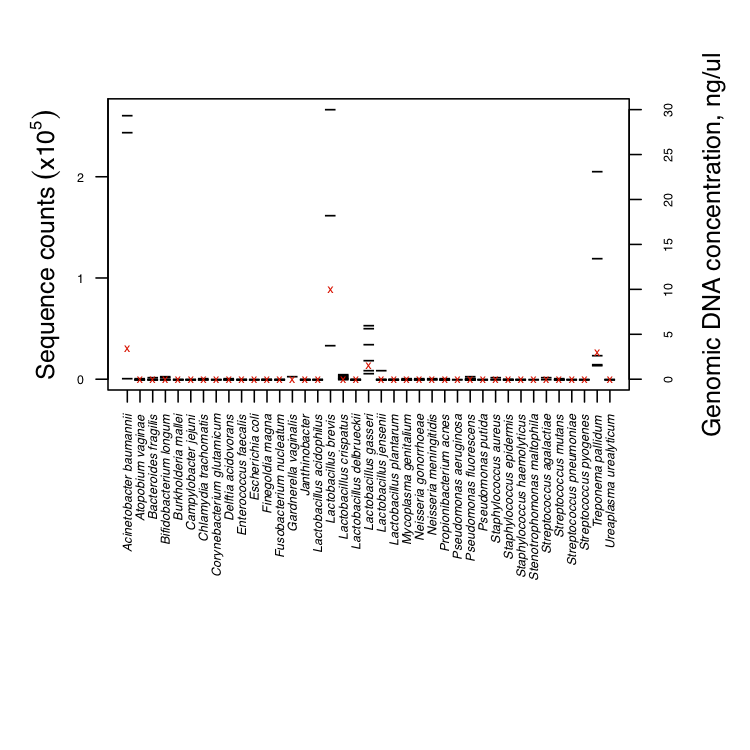


**Additional file 6: Figure S3.** Quantitative data for the SOLiD assay for simulated clinical sample D (SCD). The red crosses indicate the known concentrations of each genomic DNA (right ordinate). The horizontal lines indicate the number of sequence reads for each individual molecular probe (left ordinate). Individual bacteria are listed alphabetically across the abscissa. Some of the molecular probes for *L. crispatus* and *L. jensenii* DNAs registered positive by cross-reaction with *L. gasseri* DNA.


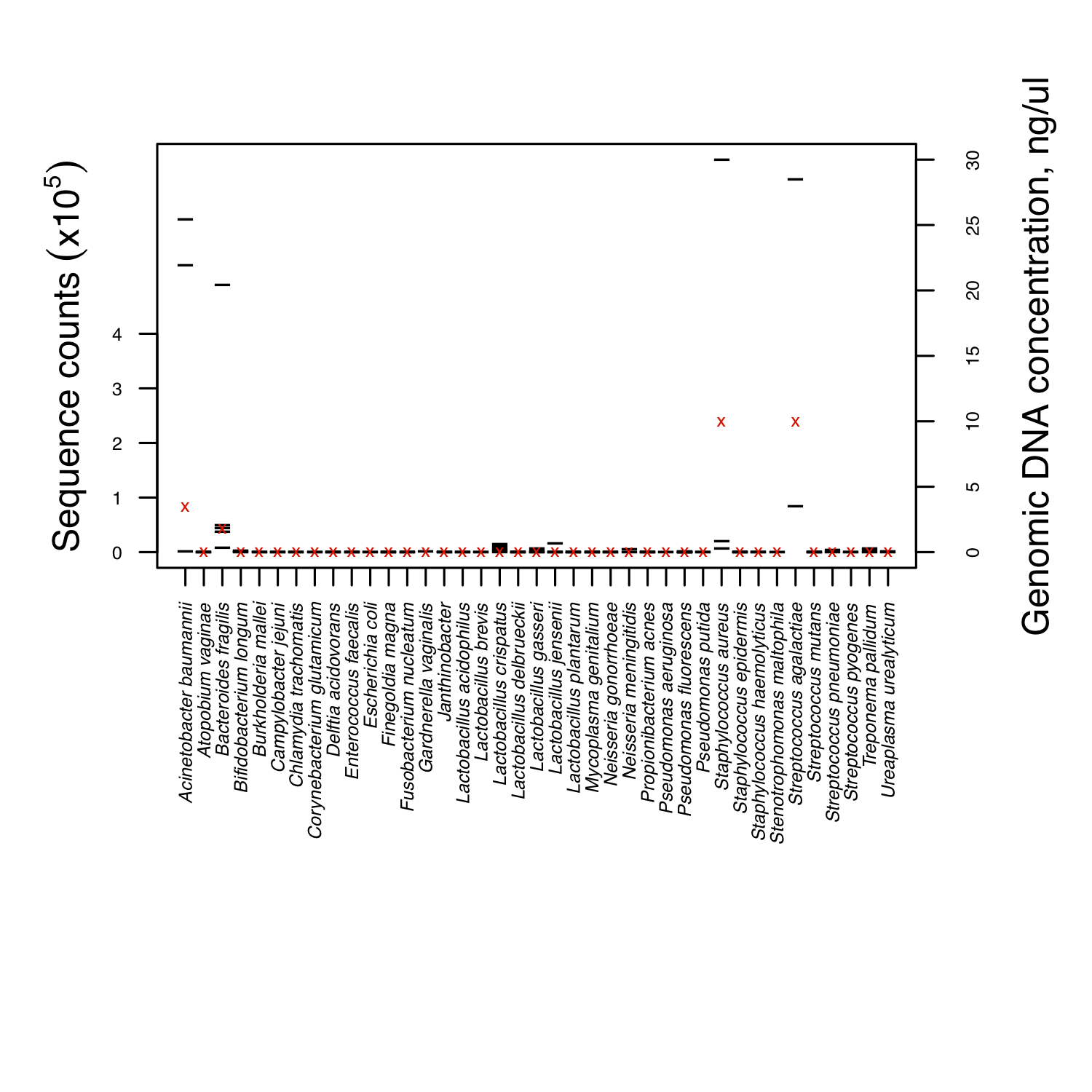


**Additional file 7: Figure S4.** Quantitative data for the SOLiD assay for simulated clinical sample E (SCE). The red crosses indicate the known concentrations of each genomic DNA (right ordinate). The horizontal lines indicate the number of sequence reads for each individual molecular probe (left ordinate). Individual bacteria are listed alphabetically across the abscissa.


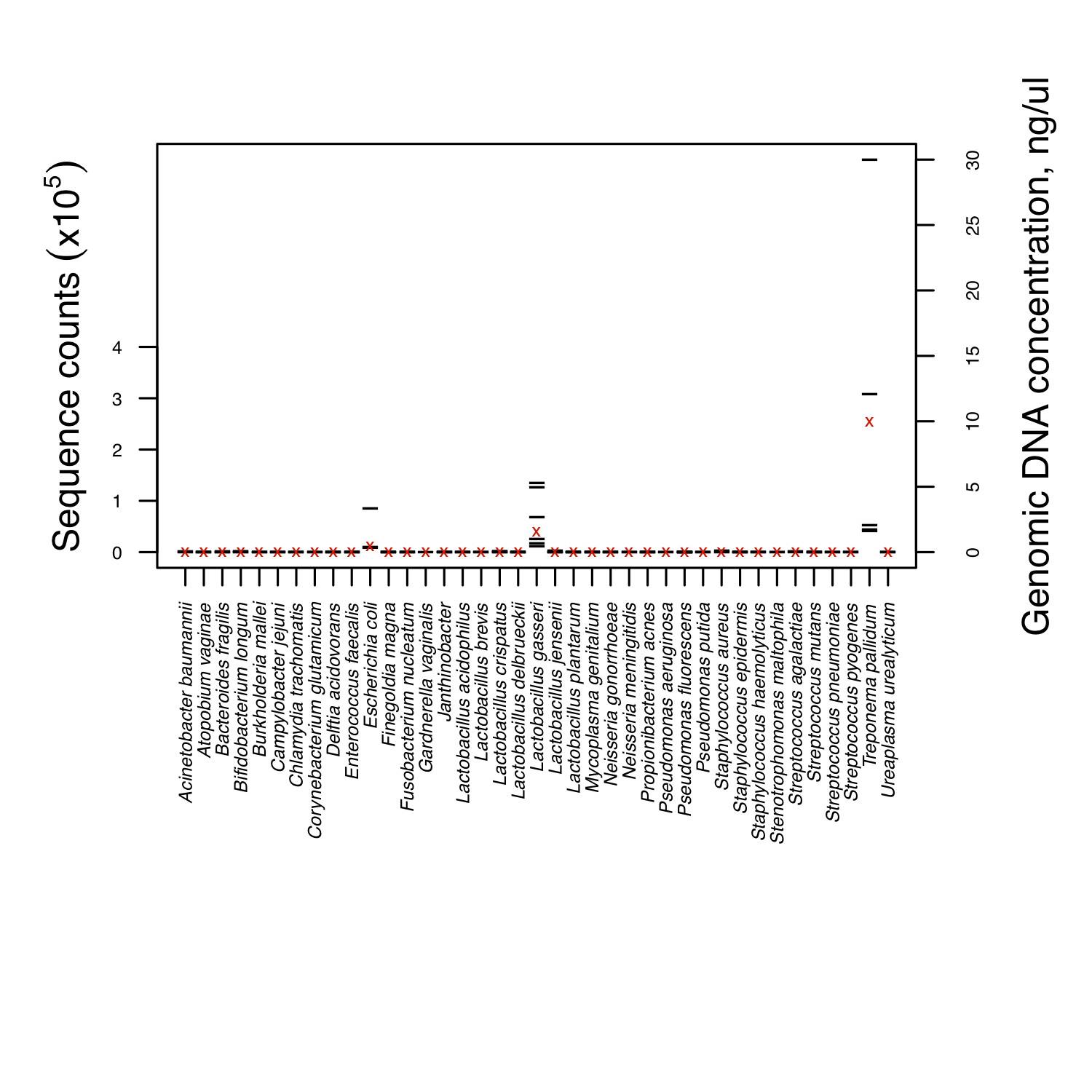

Supplement: Additional file 1 — Table S1. Amplification primers for subsequent SOLiD sequencing. Table S2. Clinical samples: comparison of BigDye-terminator reads, Tag4 fluorescent signals, and SOLiD reads. The BigDye-terminator data are from [5]. Table S3. Bacteria and the RefSeq numbers for their genome sequences. Figure S1. Quantitative data for the SOLiD assay for simulated clinical sample A (SCA). Figure S2. Quantitative data for the SOLiD assay for simulated clinical sample C (SCC). Figure S3. Quantitative data for the SOLiD assay for simulated clinical sample D (SCD). Figure S4. Quantitative data for the SOLiD assay for simulated clinical sample E (SCE). [file 1471-2180-12-29-S1.DOCX]
